# Volatile Compounds in Dry Dog Foods and Their Influence on Sensory Aromatic Profile

**DOI:** 10.3390/molecules18032646

**Published:** 2013-02-27

**Authors:** Kadri Koppel, Koushik Adhikari, Brizio Di Donfrancesco

**Affiliations:** The Sensory Analysis Center, Department of Human Nutrition, Justin Hall, Kansas State University, Manhattan, KS 66506-1407, USA; E-Mails: koushik@ksu.edu (K.A.); briziod@ksu.edu (B.D.)

**Keywords:** descriptive sensory analysis, headspace-solid phase microextraction, dog food, volatile compounds

## Abstract

The aim of this study was to determine volatile compounds in dry dog foods and their possible influence on sensory aromatic profile. Grain-free dry dog foods were compared to dry dog foods manufactured with grain, but also with different protein sources for their aromatic volatiles. Solid-phase microextraction/gas chromatography/mass spectrometry was used to determine the aromatic compounds present in the headspace of these samples. Partial Least Squares regression was performed to correlate the instrumental aromatic data with the descriptive aroma analysis data. A total of 54 aromatic compounds were tentatively identified in the dry dog food samples, with aldehydes and ketones being the most represented organic volatiles group. Grain-added products were on the average higher in total volatiles than grain-free products. Partial Least Squares regression analysis indicated possible connections with sensory aromatic profile and grain-added samples, such as rancid aroma and aldehydes, especially hexanal. The results of this study showed that dry dog foods are products with complex odor characteristics and that grain-free products are less aromatic.

## 1. Introduction

Pet food production is a part of the food production industry that has grown significantly in recent years. In 2012 more than 20 million tons of pet food was produced worldwide [1]. According to recent information collected by the American Veterinary Medical Association, there are about 70 million pet dogs in the US and that most pet owners view their pets as family members or companions [2]. Trends in human food production, such as organic, gluten-free, and natural foods are often transferred to pet food production as well [3].

Packaging and labeling play an important part in product selection off the store shelf. However, for pet food, repurchase depends on the appearance, aroma, and palatability of the product. Palatability issues are most often studied by using palatability acceptance (one-pan test) or preference tests (two-pan method) [4]. In addition aroma characteristics [5] of canned cat food and flavor characteristics [6] of dry dog food have been studied using descriptive sensory analysis methods. Both studies found pet food samples to be complex products. Volatile compounds have been studied for numerous food products that may be raw materials for pet food [7,8,9,10,11]. Pet foods are interesting objects for aromatic composition studies as their formulation is often complex. Pet foods may contain different grains (barley, oats, rice, wheat, *etc.*), meat sources (beef, chicken, pork, duck, turkey, venison, buffalo, *etc.*), animal and/or vegetable oils, micronutrients such as vitamins and minerals, antioxidants, and other additives [12]. The formulation is often extruded in case of dry foods and technologies that use gels, gravy, and meat chunks are employed in the case of wet pet food.

The aim of this study was to characterize the volatile compounds in dry dog foods and determine which volatile compounds have an effect on the sensory aromatic profile. A total of 14 samples with varying composition of protein sources as well as grain-added and grain-free samples were studied with the objective of determining if the aromatic differences were influenced by the sample formulation. To determine the influence of the aromatic compounds in the aroma perception, descriptive sensory data from a previous study [6], carried out with the same samples, was used.

## 2. Results and Discussion

### 2.1. Dry Dog Food Volatile Aromatic Composition

As shown in Table 1, Table 2, a total of 54 aromatic compounds were tentatively identified among the dry dog food samples. These aromatic compounds were grouped as: alcohols (six compounds), aldehydes (15 compounds), ketones (11 compounds), esters (one compound), sulfur compounds (one compound), pyrazines (seven compounds), furans (two compounds), alkanes (one compound), benzene derivatives (six compounds), and terpenes (four compounds).

**Table 1:** Content (µg/kg) of aroma compounds in grain-free dog food samples.

Code	Sample			GF1		GF2		GF3		GF4		GF5		GF6	
	Compound	KI (Exp)	KI (Lit)	A	S	A	S	A	S	A	S	A	S	A	S
	*Alcohols*														
A1	1-Methylcyclohexanol	N/A	(868)c	n.d.		n.d.		n.d.		n.d.		n.d.		n.d.	
A2	1-Pentanol	N/A	768a	n.d.		n.d.		n.d.		n.d.		n.d.		n.d.	
A3	2-Furanmethanol	N/A	(866)c	n.d.		n.d.		n.d.		0.08	0.02	n.d.		0.17	0.03
A4	1-Octen-3-ol	961	942c	0.18	0.08	0.46	0.34	0.61	0.13	0.15	0.05	0.51	0.12	0.23	0.03
A5	3-Propyl-2.4-pentadiene-1-ol	979	N/A	n.d.		n.d.		n.d.		n.d.		n.d.		n.d.	
A6	Borneol	1175	1158b	n.d.		n.d.		n.d.		n.d.		0.46	0.18	n.d.	
	Total alcohols			0.18		0.46		0.61		0.23		0.97		0.39	
	*Aldehydes*														
A7	Hexanal	N/A	800a	2.1	0.83	2.51	1.2	3.54	0.51	2.58	0.5	3.33	1.16	1.49	0.29
A8	Furfural	N/A	852a	0.8	0.25	n.d.		0.52	0.03	1.52	0.15	n.d.		0.25	0.02
A9	2-Methyl-2-pentenal	N/A	(808)c	n.d.		n.d.		n.d.		n.d.		n.d.		n.d.	
A10	2-Hexenal	N/A	854b	n.d.		n.d.		n.d.		n.d.		n.d.		n.d.	
A11	Heptanal	872	901a	0.14	0.04	0.23	0.12	0.26	0.04	0.15	0.01	0.34	0.12	0.14	0.03
A12	3-Methylthiopropanal	N/A	902c	n.d.		0.14	0.05	n.d.		n.d.		n.d.		n.d.	
A13	2-Heptenal	904	(951)c	n.d.		n.d.		n.d.		n.d.		n.d.		n.d.	
A14	Benzaldehyde	910	964a	2.42	1.32	4.52	1.98	4.95	0.76	3.36	0.66	5.79	1.8	4.25	0.36
A15	Octanal	977	1004a	0.24	0.04	0.34	0.15	0.35	0.03	n.d.		0.28	0.16	n.d.	
A16	2-Octenal	1010	1060b	n.d.		0.16	0.02	n.d.		n.d.		n.d.		n.d.	
A17	Nonanal	1082	1106a	0.32	0.13	0.39	0.2	0.45	0.06	0.25	0.06	0.36	0.15	0.24	0.03
A18	2-Butyl-2-octenal	1366	(1378)c	n.d.		n.d.		n.d.		n.d.		n.d.		n.d.	
A19	Benzeneacetaldehyde	1006	1043c	0.2	0.07	0.46	0.2	0.31	0.07	0.46	0.06	0.19	0.07	0.25	0.03
A20	2,6,6-Trimethyl-1-cyclohexene-1-carboxaldehyde	1208	1218c	n.d.		n.d.		n.d.		n.d.		n.d.		0.11	0.02
A21	3-Phenyl-2-propenal	1087	N/A	n.d.		n.d.		n.d.		0.23	0.06	n.d.		n.d.	
	Total aldehydes			6.21		8.76		10.4		8.55		10.28		6.72	
	*Ketones*														
A22	1-(2-Furanyl)ethanone	N/A	910c	n.d.		n.d.		n.d.		n.d.		n.d.		n.d.	
A23	6-Methyl-2-heptanone	943	(930)c	n.d.		n.d.		n.d.		n.d.		n.d.		n.d.	
A24	2.6-Octanedione	963	N/A	n.d.		n.d.		n.d.		n.d.		0.1	0.03	n.d.	
A25	6-Methyl-5-hepten-2-one	965	985c	n.d.		0.37	0.21	0.62	0.02	n.d.		0.23	0.04	n.d.	
A26	2-Heptanone	865	(898)d	n.d.		n.d.		0.18	0.06	0.11	0.03	0.21	0.08	0.15	0.02
A27	5-Methyl-2-(1-methylethyl)cyclohexanone	1159	(1137)c	n.d.		1.12	0.46	n.d.		n.d.		n.d.		n.d.	
A28	(*E,E*)-3.5-Octadien-2-one	1030	(1057)c	0.25	0.15	0.73	0.39	0.47	0.09	0.47	0.05	n.d.		n.d.	
A29	2-Nonanone	1072	1093b	n.d.		n.d.		n.d.		n.d.		n.d.		n.d.	
A30	2,2-Dihydroxy-1-phenylethanone,	1020	N/A	0.15	0.08	0.23	0.15	0.27	0.06	0.24	0.04	n.d.		n.d.	
A31	3.5-Octadien-2-one	1017	(1049)c	0.17	0.08	0.81	0.32	n.d.		0.59	0.02	1.07	0.39	n.d.	
A32	3-Octen-2-one	999	(1055)d	n.d.		n.d.		n.d.		n.d.		n.d.		n.d.	
	Total ketones			0.57		3.27		1.54		1.4		1.6		0.15	
	*Esters*														
A33	Decanoic acid, ethyl ester	1378	1394c	n.d.		n.d.		n.d.		n.d.		n.d.		n.d.	
	*Sulfur compounds*														
A34	Dimethyl disulfide	N/A	785b	n.d.		n.d.		n.d.		n.d.		n.d.		n.d.	
	*Pyrazines*														
A35	Methylpyrazine	N/A	828b	n.d.		1.66	0.68	n.d.		n.d.		n.d.		0.26	0.07
A36	2,5-Dimethylpyrazine	N/A	911c	n.d.		n.d.		0.4	0.05	0.25	0.04	0.35	0.12	0.63	0.1
A37	2,3 Dimethylpyrazine	N/A	920c	n.d.		n.d.		n.d.		n.d.		n.d.		0.17	0.02
A38	Trimethylpyrazine	926	1000b	n.d.		n.d.		n.d.		n.d.		0.3	0.13	0.31	0.05
A39	2-Ethenyl, 6-methylpyrazine	932	N/A	n.d.		n.d.		n.d.		n.d.		n.d.		0.12	0.02
A40	Tetramethylpyrazine	1025	1085c	n.d.		n.d.		n.d.		n.d.		0.73	0.22	0.48	0.05
A41	2-Ethyl, 3,5-dimethylpyrazine	1022	(1064)c	n.d.		n.d.		0.17	0.07	n.d.		n.d.		0.19	0.01
	Total pyrazines			0.00		1.66		0.57		0.25		1.38		2.16	
	*Furans*														
A42	2-butylfuran	895	N/A	n.d.		n.d.		n.d.		n.d.		n.d.		n.d.	
A43	2-Pentylfuran	994	(991)c	0.33	0.13	0.41	0.04	0.66	0.36	0.92	0.12	0.87	0.35	0.73	0.1
	Total furans			0.33		0.41		0.66		0.92		0.87		0.73	
	*Alkane*														
A44	3-Methylheptane	746	N/A	n.d.		n.d.		n.d.		n.d.		n.d.		n.d.	
	*Benzene derivatives*														
A45	Styrene	901	893b	0.48	0.34	0.53	0.38	0.7	0.43	0.52	0.27	0.6	0.36	0.65	0.28
A46	1-Ethyl-2-methyl benzene	989	(971)c	n.d.		n.d.		n.d.		n.d.		n.d.		n.d.	
A47	1,2,3-Trimethyl benzene	1003	996c	0.36	0.04	0.68	0.32	0.51	0.12	0.7	0.12	0.54	0.12	1.1	0.15
A48	Butylated hydroxytoluene	1516	N/A	0.11	0.03	n.d.		0.12	0.05	0.22	0.03	n.d.		0.1	0.03
A49	Phenol	N/A	(961)c	n.d.		n.d.		n.d.		n.d.		0.57	0.09	n.d.	
A50	1-Methyl-4-(1-methylethyl)1,4-cyclohexadiene	1069	1074c	n.d.		n.d.		n.d.		n.d.		0.55	0.17	n.d.	
	Total Benzene derivatives			0.94		1.21		1.33		1.44		2.26		1.85	
	*Terpenes*														
A51	1-(*R*)-α-Pinene	932	939a	n.d.		n.d.		n.d.		n.d.		n.d.		n.d.	
A52	Β-Pinene	989	980a	n.d.		n.d.		n.d.		n.d.		n.d.		n.d.	
A53	Limonene	1041	1030a	n.d.		0.06	0.02	n.d.		0.24	0.05	n.d.		0.1	0.04
A54	Eucalyptol	1047	1039c	n.d.		0.1	0.02	n.d.		n.d.		n.d.		n.d.	
	Total terpenes			n.d.		0.16		n.d.		0.24		n.d.		0.1	
	Total			8.24		15.94		15.09		13.03		17.37		12.12	

a-[13]; b-[14]; c-[15]; () different column; d-[10]; n.d.-not detected; N/A-not available; A-average; S-standard deviation.

**Table 2:** Content (µg/kg) of aroma compounds in grain-added dog food samples.

Code	Sample			GA1		GA2		GA3		GA4		GA5		GA6		GA7		GA8	
	Compound	KI (Exp)	KI (Lit)	A	S	A	S	A	S	A	S	A	S	A	S	A	S	A	S
	*Alcohols*																		
A1	1-Methylcyclohexanol	N/A	(868)c	n.d.		n.d.		n.d.		0.9	0.02	1.43	0.62	n.d.		n.d.		0.72	0.08
A2	1-Pentanol	N/A	768a	n.d.		n.d.		n.d.		n.d.		n.d.		n.d.		2.26	0.14	n.d.	
A3	2-Furanmethanol	N/A	(866)c	n.d.		n.d.		n.d.		n.d.		n.d.		0.34	0.04	n.d.		n.d.	
A4	1-Octen-3-ol	961	942c	0.98	0.14	0.36	0.08	0.81	0.04	0.24	0.06	0.49	0.35	0.12	0.02	1.48	0.18	0.35	0.08
A5	3-Propyl-2.4-pentadiene-1-ol	979	N/A	n.d.		n.d.		0.27	0.04	n.d.		0.11	0.06	n.d.		0.93	0.1	n.d.	
A6	Borneol	1175	1158b	n.d.		n.d.		n.d.		n.d.		n.d.		n.d.		n.d.		n.d.	
	Total alcohols			0.98		0.36		1.08		1.14		2.03		0.46		4.66		1.07	
	*Aldehydes*																		
A7	Hexanal	N/A	800a	10.79	0.87	4.25	0.12	9.24	1.17	4.8	0.14	10.15	5.27	1.26	0.26	9.17	0.88	2.91	0.47
A8	Furfural	N/A	852a	0.51	0.08	0.2	0.06	n.d.		0.81	0.03	0.53	0.19	1.41	0.07	n.d.		0.24	0.05
A9	2-Methyl-2-pentenal	N/A	(808)c	n.d.		n.d.		n.d.		n.d.		n.d.		n.d.		0.53	0.05	n.d.	
A10	2-Hexenal	N/A	854b	n.d.		n.d.		n.d.		n.d.		0.18	0.07	n.d.		n.d.		n.d.	
A11	Heptanal	872	901a	0.96	0.09	0.39	0.02	0.56	0.13	0.23	0.02	0.57	0.3	0.14	0.03	0.86	0.11	0.21	0.05
A12	3-Methylthiopropanal	N/A	902c	n.d.		n.d.		n.d.		n.d.		n.d.		0.16	0.03	n.d.		n.d.	
A13	2-Heptenal	904	(951)c	n.d.		0.56	0.14	n.d.		n.d.		n.d.		n.d.		n.d.		n.d.	
A14	Benzaldehyde	910	964a	4.97	0.17	3.76	0.06	5.33	0.62	2.93	0.25	5.5	2.68	3.2	0.09	8.02	0.73	2.58	0.39
A15	Octanal	977	1004a	1.2	0.2	0.28	0.04	0.56	0.07	0.27	0.02	0.56	0.29	0.12	0.04	0.96	0.07	0.2	0.06
A16	2-Octenal	1010	1060b	0.32	0.09	n.d.		0.16	0.05	n.d.		0.44	0.27	n.d.		n.d.		n.d.	
A17	Nonanal	1082	1106a	1.73	0.24	0.44	0.03	0.71	0.15	0.44	0.05	0.85	0.52	0.27	0.05	1.1	0.14	0.41	0.09
A18	2-Butyl-2-octenal	1366	(1378)c	0.17	0.03	n.d.		0.18	0.05	n.d.		0.15	0.09	n.d.		0.24	0.07	n.d.	
A19	Benzeneacetaldehyde	1006	1043c	0.34	0.06	n.d.		0.2	0.04	0.19	0.01	0.12	0.05	0.48	0.04	0.19	0.00	0.09	0.03
A20	2,6,6-Trimethyl-1-cyclohexene-1-carboxaldehyde	1208	1218c	n.d.		n.d.		n.d.		n.d.		n.d.		n.d.		n.d.		n.d.	
A21	3-Phenyl-2-propenal	1087	N/A	n.d.		n.d.		n.d.		n.d.		n.d.		n.d.		n.d.		n.d.	
	Total aldehydes			20.98		9.89		16.94		9.67		19.04		7.03		21.07		6.64	
	*Ketones*																		
A22	1-(2-Furanyl)ethanone	N/A	910c	n.d.		n.d.		n.d.		n.d.		n.d.		0.18	0.01	n.d.		n.d.	
A23	6-Methyl-2-heptanone	943	(930)c	n.d.		n.d.		n.d.		n.d.		n.d.		n.d.		0.11	0.02	n.d.	
A24	2.6-Octanedione	963	N/A	n.d.		0.05	0.03	0.09	0.06	n.d.		n.d.		n.d.		n.d.		0.1	0.05
A25	6-Methyl-5-hepten-2-one	965	985c	n.d.		n.d.		0.19	0.04	n.d.		0.58	0.31	n.d.		n.d.		n.d.	
A26	2-Heptanone	865	(898)d	0.2	0.02	0.14	0.03	0.4	0.02	n.d.		0.43	0.23	0.09	0.01	0.68	0.07	0.14	0.04
A27	5-Methyl-2-(1-methylethyl) cyclohexanone	1159	(1137)c	n.d.		n.d.		n.d.		n.d.		n.d.		n.d.		n.d.		n.d.	
A28	(*E,E*)-3.5-Octadien-2-one	1030	(1057)c	0.69	0.1	n.d.		0.48	0.05	0.59	0.04	1.55	0.94	n.d.		1.6	0.33	0.33	0.04
A29	2-Nonanone	1072	1093b	n.d.		n.d.		0.16	0.02	n.d.		n.d.		n.d.		0.17	0.02	0.03	0.01
A30	2,2-Dihydroxy-1-phenylethanone	1020	N/A	0.16	0.03	n.d.		n.d.		0.26	0.02	n.d.		0.14	0.04	0.46	0.03	0.18	0.03
A31	3.5-Octadien-2-one	1017	(1049)c	0.25	0.03	n.d.		n.d.		0.6	0.07	1.88	0.68	n.d.		0.95	0.04	0.25	0.05
A32	3-Octen-2-one	999	(1055)d	n.d.		n.d.		n.d.		n.d.		0.99	0.54	n.d.		0.84	0.11	n.d.	
	Total ketones			1.3		0.2		1.34		1.45		5.43		0.41		4.8		1.03	
	*Esters*																		
A33	Decanoic acid, ethyl ester	1378	1394c	n.d.		n.d.		n.d.		n.d.		n.d.		n.d.		0.26	0.02	n.d.	
	*Sulfur compounds*																		
A34	Dimethyl disulfide	N/A	785b	n.d.		n.d.		n.d.		n.d.		n.d.		0.45	0.09	n.d.		n.d.	
	*Pyrazines*																		
A35	Methyl pyrazine	N/A	828b	n.d.		n.d.		n.d.		n.d.		n.d.		n.d.		n.d.		n.d.	
A36	2,5-Dimethyl pyrazine	N/A	911c	n.d.		3.85	0.15	0.25	0.08	n.d.		n.d.		0.43	0.11	0.21	0.05	0.37	0.28
A37	2,3 Dimethyl pyrazine	N/A	920c	n.d.		n.d.		n.d.		n.d.		n.d.		n.d.		n.d.		n.d.	
A38	Trimethyl pyrazine	926	1000b	n.d.		n.d.		n.d.		n.d.		n.d.		n.d.		n.d.		n.d.	
A39	2-Ethenyl, 6-methyl pyrazine	932	N/A	n.d.		n.d.		n.d.		n.d.		n.d.		n.d.		n.d.		n.d.	
A40	Tetramethyl pyrazine	1025	1085c	n.d.		n.d.		n.d.		n.d.		n.d.		n.d.		n.d.		n.d.	
A41	2-Ethyl, 3,5-dimethyl pyrazine	1022	(1064)c	n.d.		0.32	0.04	0.16	0.02	n.d.		n.d.		0.19	0.02	n.d.		0.2	0.17
	Total pyrazines			n.d.		4.17		0.41		n.d.		n.d.		0.63		0.21		0.57	
	*Furans*																		
A42	2-butylfuran	895	N/A	n.d.		n.d.		n.d.		n.d.		0.15	0.08	n.d.		n.d.		n.d.	
A43	2-Pentylfuran	994	(991)c	2.19	0.32	0.86	0.07	1.69	0.25	0.64	0.18	2.45	1.19	0.42	0.03	3.17	0.49	0.58	0.22
	Total furans			2.19		0.86		1.69		0.64		2.59		0.42		3.17		0.58	
	*Alkane*																		
A44	3-Methylheptane	746	N/A	n.d.		n.d.		n.d.		n.d.		0.48	0.25	n.d.		n.d.		n.d.	
	*Benzene derivatives*																		
A45	Styrene	901	893b	0.48	0.28	2.83	3.86	0.55	0.17	0.68	0.26	0.41	0.3	0.43	0.26	0.62	0.28	0.34	0.18
A46	1-Ethyl-2-methylbenzene	989	(971)c	n.d.		0.1	0.01	n.d.		n.d.		n.d.		n.d.		n.d.		n.d.	
A47	1,2,3-Trimethylbenzene	1003	996c	0.49	0.11	0.73	0.01	1.07	0.19	0.34	0.11	0.27	0.15	0.41	0.09	6.55	9.78	0.46	0.03
A48	Butylated hydroxytoluene	1516	N/A	0.05	0.01	0.09	0.03	0.12	0.04	0.06	0.02	0.1	0.04	0.37	0.08	n.d.		0.05	0.03
A49	Phenol	N/A	(961)c	n.d.		n.d.		n.d.		n.d.		n.d.		n.d.		n.d.		n.d.	
A50	1-Methyl-4-(1-methylethyl)1,4-cyclohexadiene	1069	1074c	n.d.		n.d.		n.d.		n.d.		n.d.		n.d.		n.d.		n.d.	
	Total Benzene derivatives			1.02		3.74		1.74		1.08		0.77		1.2		7.16		0.84	
	*Terpenes*																		
A51	1(*R*)-α-Pinene	932	939a	n.d.		n.d.		0.13	0.02	n.d.		n.d.		n.d.		n.d.		n.d.	
A52	β-Pinene	989	980a	n.d.		0.16	0.02	n.d.		n.d.		n.d.		n.d.		n.d.		n.d.	
A53	Limonene	1041	1030a	n.d.		n.d.		n.d.		0.16	0.01	n.d.		n.d.		n.d.		n.d.	
A54	Eucalyptol	1047	1039c	n.d.		0.24	0.02	n.d.		n.d.		n.d.		n.d.		n.d.		n.d.	
	Total terpenes			n.d.		0.4		0.13		0.16		n.d.		n.d.		n.d.		n.d.	
	Total			26.48		19.63		23.33		14.14		30.35		10.6		41.34		10.73	

a-[13]; b-[14]; c-[15]; () different column; d-[10]; n.d.-not detected; N/A-not available; A-average; S-standard deviation.

Total concentration of volatiles was higher in the grain-added samples (10.60–41.34 µg/kg, average 22.07 µg/kg) than the grain-free samples (8.24–17.37 µg/kg, average 13.63 µg/kg). The addition of different grains such as wheat, barley, corn, rice, or sorghum in the formulation of dry dog food might have influenced the total volatile concentrations. These grains, if not fractionated, usually contain protein such as gluten, carbohydrates such as starch, lipids, water, fiber, B-vitamins, and minerals [16]. According to Busko *et al.* [8] rice has the lowest levels of volatile organic compounds, while durum wheat has the highest. In this study it was found that the sample manufactured with only one type of grain – rice – had the second highest total volatiles concentration of the samples studied (sample GA5, 30.35 µg/kg). This result indicates other ingredients in the formulation and reactions during processing such as Maillard browning and other oxidative reactions are important for development of the total volatiles concentrations.

In general more than 50% of the total amount of volatile compounds in all the samples was aldehydes (Table 1, Table 2). The aldehyde content varied from 6.21–10.40 µg/kg in the grain free and from 6.64–21.07 µg/kg in the grain added samples. Animal fat, especially chicken fat, is very common in pet food formulations [17] (Table 3) and aldehydes are one of the most common products resulting from lipid/fat oxidation reactions [18]. Moreover, it has been shown that the drying process in food containing lipids can influence the amount of compounds such as aldehydes and ketones. Thus, it influences the flavor and in certain products these compounds can present an indicator of oxidized flavor [19]. Aldehydes, together with alcohols and ketones, are some of the main volatile compounds present in most of the common cereal grains. Aldehydes play an important role in cereal quality aspects and, with a low odor threshold value, have a considerable impact on the aroma of cereal products. In a recent study aldehydes were detected as the most abundant group of volatiles in grain products [20]. The most abundant aldehyde detected was benzaldehyde, which is commonly known as almond oil. The second most abundant aldehyde was hexanal, often contributing to the green odor notes in foods. Both of these aldehydes have been reported in a variety of products including ham [9], sausages [21], turkey breast [7], different grain products [11,22], and dairy products [19]. In addition to hexanal, heptanal, octanal, and nonanal which are common lipid oxidation products, were found in most samples. Furfural was found in four of the grain-free and six of the grain-added samples. Furfural is an oxygenated furan which usually forms during the Maillard reaction and has caramel-like and fruity characteristics [18].

All seven pyrazine compounds were tentatively identified in one of the grain-free samples, GF6. Pyrazines are organic compounds that contain nitrogen and often are characterized by nutty and roasted aromatics, and these compounds are formed during the Maillard reaction [18]. 

Two volatile compounds, styrene and 1,2,3-trimethylbenzene, were detected in all of the samples. The origin of these compounds is unclear as according to previous studies by [23] and [24] styrene is found naturally in many foods such as almonds, beef and wheat. However, styrene and benzaldehyde (mentioned above) may also migrate from plastic packaging materials and are considered pollutants [25,26]. Another study, [27] found that trimethylbenzene is very likely an environmental pollutant, migrating from packaging material into cheese products due to its highly lipophilic properties. A similar process may well take place in case of dry dog foods, which are usually packaged in multi-layered plastic bags and have a matrix that contains fat.

**Table 3:** Sample codes, nutrient contents, and ingredients.

Number	Code	Protein, %	Fat, %	Ingredients *
1	GF1	26.5	17.1	Chicken, pea protein concentrate, potato starch, dried potato, chicken meal, chicken fat, dried beet pulp, flaxseed, chicken liver flavor, powdered cellulose, lactic acid, cranberries, apples, peas, carrots, broccoli, iodized salt, choline chloride, vitamins, potassium chloride, minerals, taurine, beta-carotene, phosphoric acid, rosemary extract.
2	GF2	27	17	De-boned turkey, potato, whole dried egg, pea, flaxseed, apple, canola oil, natural flavor, coconut oil, tomato, salmon, de-boned duck, sundried alfalfa, carrots, pumpkin, bananas, blueberries, cranberries, raspberries, blackberries, papaya, pineapple, grapefruit, lentil beans, broccoli, spinach, cottage cheese, alfalfa sprouts, dried kelp, lecithin, vitamins, minerals, taurine, DL-methionine, L-lysine, algae extract, chicory extract, *Lactobacillus acidophilus*, *Lactobacillus casei*, *Enterococcus faecium*, *Bifidobacterium termophilum*, dried *Aspergillus niger* fermentation extract, dried *Aspergillus oryzae* fermentation extract, yeast extract, *Yucca schidigera* extract, marigold extract, parsley, peppermint, green tea extract, L-carnitine, dried rosemary.
3	GF3	33	10	Deboned turkey, turkey meal, chicken meal, potatoes, peas, dried ground potatoes, pea fiber, tomato pomace, chicken fat, chicken liver, natural chicken flavor, ground flaxseed, salmon oil, carrots, sweet potatoes, kale, broccoli, spinach, parsley, apples, blueberries, vitamins, minerals, choline chloride, mixed tocopherols added to preserve freshness, glucosamine hydrochloride, chondroitin sulfate, taurine, chicory root extract, *Yucca schidigera* extract, dried *Lactobacillus plantarum *fermentation product, dried *Enterococcus faecium* fermentation product, dried *Lactobacillus casei* fermentation product, dried *Lactobacillus acidophilus* fermentation product, rosemary extract.
4	GF4	20	10	Sweet potatoes, venison, potato protein, pea protein, canola oil, dicalcium phosphate, potato fiber, flaxseed, natural flavor, choline chloride, taurine, natural mixed tocopherols, vitamins, minerals.
5	GF5	41	20	Ocean fish meal, beef, potatoes, pea protein, canola oil, dried eggs, peas, tomato pomace, natural flavor, potassium chloride, choline chloride, salmon oil, dried chicory root, taurine, parsley flakes, pumpkin meal, almond oil, sesame oil, Yucca schidigera extract, thyme, blueberries, cranberries, carrots, broccoli, vitamins, minerals, rosemary extract
6	GF6	27	12	Chicken, potatoes, chicken meal, pea protein, peas, sweet potatoes, poultry fat (preserved with mixed tocopherols), apples, pumpkin, natural flavor, tapioca starch, tomato pomace, salt, potassium chloride, choline chloride, vitamins, minerals, citric acid (used as a preservative), *Yucca schidigera extract*, rosemary extract.
7	GA1	24.7	16	Chicken by-product meal, whole grain corn, brewers rice, powdered cellulose, soybean mill run, animal fat, soybean oil, lactic acid, chicken liver flavor, flaxseed, potassium chloride, iodized salt, choline chloride, vitamin E supplement, vitamins, taurine, minerals, phosphoric acid, beta-carotene, rosemary extract.
8	GA2	28	16	Beef, soybean meal, soy flour, animal fat, brewers rice, soy protein concentrate, corn gluten meal, ground yellow corn, glycerin, poultry by-product meal, ground wheat, animal digest, pearled barley, calcium carbonate, calcium phosphate, salt, grilled sirloin steak flavor, dried green beans, dried potatoes, sulfur, Vitamin E supplement, choline chloride, zinc sulfate, ferrous sulfate, added color (Red 40, Blue 2, Yellow 5, Yellow 6), niacin, wheat flour, potassium chloride, L-Lysine monohydrochloride, vitamins, minerals, garlic oil, C-5900
9	GA3	25	14	Chicken meal, ground rice, pearled barley, poultry fat, natural flavor, tomato pomace, salt, potassium chloride, minerals, yeast culture, choline chloride, *Yucca schidigera* extract, rosemary extract, citric acid (preservative).
10	GA4	19.5	15.5	Whole grain corn, chicken by-product meal, animal fat, soybean mill run, flaxseed, chicken liver flavor, lactic acid, corn gluten meal, potassium chloride, L-lysine, choline chloride, vitamin E supplement, iodized salt, vitamins, calcium carbonate, dicalcium phosphate, minerals, L-tryptophan, taurine, glucosamine hydrochloride, L-carnitine, chondroitin sulfate, phosphoric acid, beta-carotene, rosemary extract.
11	GA5	20	12	Ground rice, deboned duck, rice protein concentrate, sunflower oil, flaxseed, dicalcium phosphate, natural duck flavor, vitamins, minerals, choline chloride, taurine.
12	GA6	27	16	Chicken meal, rice, brown rice, corn gluten meal, chicken fat, barley, natural chicken flavor, dried beet pulp (sugar removed), rice flour, dried egg product, anchovy oil, dried brewers yeast, potassium chloride, flaxseed, calcium carbonate, fructo-oligosaccharides (FOS), salt, choline chloride, sodium tripolyphosphate, DL-methionine, vitamins, taurine, salmon meal, trace minerals, glucosamine hydrochloride, tea (green tea extract), L-carnitine, chondroitin sulfate, marigold extract (*Tageteserecta l*.), rosemary extract.
13	GA7	23	14	Salmon, brewers rice, ground whole grain sorghum, potato, ground whole grain barley, chicken meal, fish meal, chicken fat, dried egg product, dried beet pulp, natural flavor, brewers dried yeast, potassium chloride, salt, sodium hexametaphosphate, calcium carbonate, DL-methionine, choline chloride, fructo oligosaccharides, minerals, vitamins, beta-carotene, rosemary extract.
14	GA8	18	7.9	Turkey, chicken, barley, brown rice, potato, rice, pea fiber, chicken meal, herring, natural flavors, chicken fat, flaxseed, apple, carrot, herring oil, sunflower oil, egg ,cottage cheese, alfalfa sprouts, pumpkin, dried chicory root, L-carnitine ,vitamins, minerals, direct fed microbials (dried *Lactobacillus acidophilus *fermentation product, dried *Lactobacillus casei* fermentation product, dried *Enterococcus faecium* fermentation product), lecithin, rosemary extract.

* Vitamins and minerals have not been listed in detail.

Antioxidants are added to most processed and packaged foods to prevent oxidation processes. Butylated hydroxytoluene (BHT), butylated hydroxyanisole (BHA), tocopherols, and organic acids, as well as spices and plant products are among the most common antioxidants in pet foods [28]. In this study, BHT was detected in nine of the 12 samples. In addition to possible incorporation of BHT into pet food formulation, according to [29] BHT may also migrate into foods from packaging materials and other raw materials. Minor amounts of alphapinene, betapinene, limonene, and eucalyptol were detected in some samples. These are likely to originate from rosemary and other extracts added to the formulations (Table 3).

### 2.2. Partial Least Squares Regression

**Figure 1:**
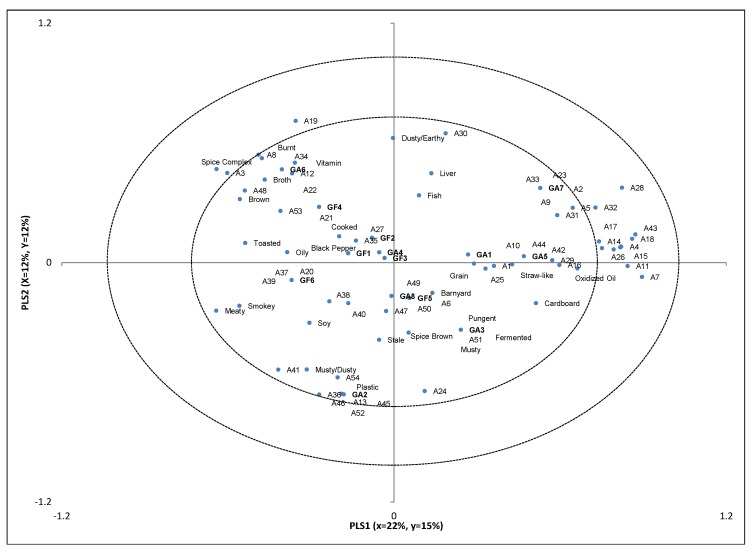
Partial Least Squares Regression factors 1 and 2.

**Figure 2:**
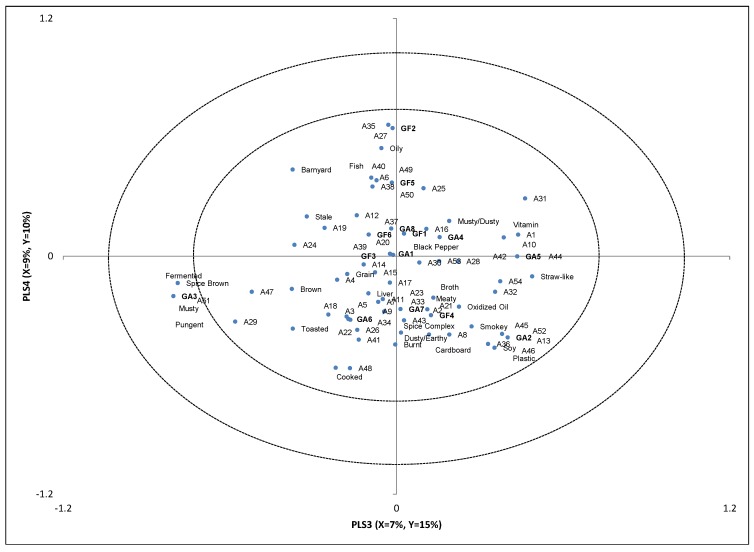
Partial Least Squares Regression factors 3 and 4.

Figure 1, Figure 2 show the partial least squares regression maps where the sensory aroma attributes were correlated with the instrumental aromatic profile. The percentages explained by the first four partial least squares factors were low, 50% of volatile compound data variation explained 52% of descriptive sensory analysis data variation. Overall more associations with volatile compounds were found among grain-added samples. This might be because of the presence of more volatiles in the grain-added samples.

There were several correlations, although weak, between volatile compounds and descriptive sensory attributes. Hexanal (A7) and other compounds such as heptanal (A11), benzaldehyde (A14), octanal (A15) 2-octenal (A16), nonanal (A17), 2-butyl-2-octenal (A18), 2-heptanone (A26), and 2-nonanone (A29) seemed to be related to oxidized oil aromatics and less with straw-like and cardboard aromatics and associated with sample GA5. Hexanal has been reported to have a green, but also tallow and fat odor [15]. Lee *et al.* [30] also reported hexanal and other aldehyde and ketone compounds associations with rancid flavors. While for most food products rancid odor may be not a wanted characteristic, for dry dog food this may not be the case. Dogs may or may not refuse a food because of rancid odor. However, decisions about buying and serving pet food are usually made by the owner, who may not find the rancid odor acceptable.

Plastic and musty/dusty aromatics were associated with styrene (A45), 2-heptenal (A13), β-pinene (A52), eucalyptol (A54), and 1-ethyl-2-methylbenzene (A46), which seemed to be related with sample GA2. In fact, A46, A52, and A13 were detected only in sample GA2, perhaps originating from one of the ingredients. Styrene, as mentioned earlier, has been found to originate from packaging materials, and has balsamic or gasoline aromatics, while β-pinene has been reported to have pine, resin, and turpentine aromatics [14].

Burnt, spice complex, and vitamin aromatics were associated with 2-furanmethanol (A3), furfural (A8), benzene acetaldehyde (A19), 1-(2-furanyl)ethanone (A22), dimethyl disulfide (A34), and 3-methylthiopropanal (A12), which were associated with sample GA6. Furanmethanol has been reported as having burnt aromatics [15], and this was also found by descriptive analysis. Dimethyl disulfide (A34) has been reported as having vegetable notes such as cabbage and onion [15], which could be related to brothy aromatics (defined as the aromatic sensation associated with boiled meat, soup, or stock, and usually referenced by chicken broth). Benzeneacetaldehyde (A19), furfural (A8), and 1-(2-furanyl)ethanone (A22) may have some sweet aromatics [15] however sweet aromatics were not detected in descriptive sensory analysis. The reason for this may be that the detection thresholds for these compounds may have been higher than the levels detected in the samples, although according to [22] the thresholds were lower than the quantities detected. This suggests sweet aromatics may have been masked by other characteristics such as barnyard or were evaluated as part of a different attribute such as straw-like, which was defined as somewhat sweet, dry, slightly dusty aromatics with the absence of green; associated with dry grain stems.

According to Figure 2 fermented, musty, and pungent aromatics were associated with 1(*R*)-α-pinene (A51) and seemed to be related with sample GA3. 1(*R*)-α-pinene is a pine aromatic [15], which could be related to musty and pungent aromatics detected by the descriptive sensory analysis.

The only association found between grain-free samples and volatile compounds was sample GF2 and GF5 association with oily, fish, and barnyard aromatics. Methylpyrazine (A35) and 5-methyl-2-(1-methyl)ethylcyclohexanone (A27) were found in sample GF2. Methylpyrazine (A35) carries popcorn aromatics while A27 has been characterized as fresh, green, minty, and woody [15]. Phenol (A49), borneol (A6), trimethylpyrazine (A38), 1-methyl-4-(1-methylethyl)-cyclohexadiene (A50), 6-methyl-5-hepten-2-one (A25) and tetramethylpyrazine (A40) were found in sample GF5. These compounds have a variety of odor characteristics from musty to fruity. There does not seem to be a direct relationship between volatile compounds and the sensory attributes. It may be that a combination of compounds rather than a single compound is responsible for these sensory characteristics.

## 3. Experimental

### 3.1. Samples

Overall 14 commercial dry dog food samples were purchased in the Manhattan, KS area (Table 3). One of the samples (GA2) was composed of three different kibbles. The samples included grain-free samples (GF1, GF2, GF3, GF4, GF5, and GF6), a sample for oral hygiene maintenance (GA1), food for pre-adolescent (GA7) and older (GA4) canines, as well as samples that contained probiotics (GF2, GF3, and GA8). The samples varied in protein source used in the formulation from poultry, turkey, and duck to pork, beef, venison, lamb, herring, and salmon. All sample lots were checked for recalls and were within their expiration dates.

### 3.2. Extraction Procedure of Volatile Aroma Compounds

The extraction method chosen for studying the aroma profile in the dry dog foods was headspace-solid phase microextraction (HS-SPME). The samples were ground in a pestle and mortar, 0.5 g was weighed in a 10 mL screw-cap vial equipped with a polytetrafluoroethylene/silicone septum. Exactly 0.48 mL distilled water was added to the ground sample in the vial. According to [31] in SPME analysis water or other surface-active compounds should be added to solid samples to improve the transport of compounds from the sample to the gaseous phase. The internal standard was 0.02 mL 1,3-dichlorobenzene dissolved in hexane (mixture of isomers, optima grade, Fisher Scientific; Pittsburgh, PA, USA), with final concentration in the sample of 40 µg/kg. The vials were equilibrated for 10 min at 40 °C in the autosampler (Pal system, model CombiPal, CTC Analytics, Zwingen, Switzerland) and agitated at 250 rpm. After the equilibration, a 50/30 µm divinylbenzene/carboxen/polydimethyl-siloxane fiber was exposed to the sample headspace for 30 min at 40 °C. The fiber was chosen for its high capacity of trapping compounds in food products [32]. After sampling, the analytes were desorbed from the SPME fiber coating to the injection port of gas chromatography (GC) at 270 °C for 3 min in splitless mode.

### 3.3. Chromatographic Analyses

The isolation, tentative identification, and semi-quantification of the volatile compounds were performed on a gas chromatograph (Varian GC CP3800; Varian Inc., Walnut Creek, CA, USA), coupled with a Varian mass spectrometer (MS) detector (Saturn 2000). The GC-MS system was equipped with an RTX-5MS (Crossbond® 5% diphenyl/95% dimethyl polysiloxane) column (Restek, U.S., Bellefonte, PA, USA; 30 m × 0.25 mm × 0.25 µm film thickness). The initial temperature of the column was 40 °C and was held at that temperature for 4 min; the temperature was then increased by 5 °C per min to 260 °C, and held at this temperature for 7 min. All samples were analyzed in triplicates.

Most of the compounds were identified using two different analytical methods: (1) mass spectra and (2) Kovats indices (NIST/EPA/NIH Mass Spectral Library, Version 2.0, 2005). Identification was considered tentative when it was based on only mass spectral data. The retention times for a C7-C40 saturated alkane mix (Supelco Analytical, Bellefonte, PA, USA) was used to determine experimental Kovats indices for the volatile compounds detected.

### 3.4. Descriptive Analysis Data for Regression

During the first part of this study, descriptive sensory analysis using the modified flavor profile consensus method was carried out to determine the main attributes and references to evaluate dry dog foods and study differences among specialty products [6]. The 27 aromatic attributes that were used in the evaluation and included in the data analysis of the samples, were: barnyard, black pepper, broth, brown, burnt, cardboard, cooked, dusty/earthy, fermented, fish, grain, liver, meaty, musty, musty/dusty, oily, oxidized oil, plastic, pungent, soy, smoky, spice brown, spice complex, stale, straw-like, toasted, and vitamin. These aroma attributes were evaluated on a 15-point scale with 0.5 increments and references provided for different scale points. The evaluation was carried out by five highly trained panelists with at least 1,000 h of experience in evaluating a variety of food products. The panelists were asked to evaluate the aroma of the products orthonasally (from a sniffing glass covered with a watch glass, amount of sample 3 g). The panelists were provided moist cloths to help eliminate aromas from their airways in between the sample evaluation.

### 3.5. Data Analysis

Partial least square regression (PLSR) study is a multivariate statistical technique that has been used by several researchers for creating external preference maps for determining relationships between descriptive data (X-matrix) and consumer acceptability data (Y-matrix) [33]. PLSR can be used to correlate the instrumental volatile data (X-matrix) and descriptive sensory data (Y-matrix) [30], which was done in this study. This procedure was carried out in our study using the Unscrambler version 10.2 (Camo Software; Oslo, Norway).

## 4. Conclusions

Up to 54 different aromatic compounds were tentatively identified and semi-quantified in six samples of grain-free and eight samples of grain-added dry dog foods. The concentration of the total volatile compounds was higher in the grain-added samples. This was mainly caused by a high concentration of aldehydes in the grain-added samples. Overall aldehydes were the most abundant group of volatiles found in dry dog foods. There were some associations found between volatile compounds and sensory analysis descriptors, such as aldehydes and rancid aromatics and benzene derivatives and plastic aromatics. The results of this study showed that dry dog foods, as are other processed foods, are products with complex odor characteristics. Looking at odor characteristics associations with consumer and dog acceptability studies would be of interest for future studies.
